# Rearrangements by the Metropolitan Asylums Board

**Published:** 1915-02-06

**Authors:** 


### Rearrangements by the Metropolitan Asylums Board.

The Metropolitan Asylums Board have found it necessary, owing to the war and the prevalence of scarlet fever and diphtheria in the Metropolis, to make a number of alterations in their hospital accommodation. For instance, the Board have now decided further to utilise the Goldie Leigh Homes, which they have taken over from the Woolwich Board of Guardians. The only children who have up to the present been received at this institution were suffering from ringworm. Dr. Galloway, the consulting physician for skin diseases, has, however, recommended that as soon as possible the skin cases which, on the closing of the Park Hospital as an institution for sick and debilitated children, were sent to Queen Mary's Hospital should be transferred to Goldie Leigh Homes, where all cases of skin diseases should be treated. It had not been found practicable to carry out these proposals, as, in the opinion of Dr. Galloway, the existing arrangements for the medical care of the children were not adequate. At present, just as before the institution was taken over from the Woolwich Guardians, the homes are visited three times a week, and at other times, if necessary, by Dr. W. E. Boulter, the medical superintendent of the Woolwich Infirmary, who received £50 per annum for this work. Dr. Galloway advised the managers to appoint a young local medical man, who would be able and willing to devote the time necessary for regularly visiting the institution to see that the treatment was properly carried out. He would have to visit the institution five days a week, and attend on urgent cases of sickness, acting under the direction of the consulting physician. He would have to give anæsthetics, and to carry out such pathological, bacteriological, and microscopical investigations as might be necessary. Emphasis should, Dr. Galloway added, be laid on the fact that the Board would expect the duties to be carried out "so that the cases are treated and examined in a workmanlike manner, using the scientific methods necessary for the proper care of children." The Board decided to adopt the suggestion made by Dr. Galloway, deciding, for several reasons, to make full use as soon as possible of the vacant accommodation at the institution. The salary attached to the office will be £150 per annum, but in the meantime arrangements have been made with Mr. C. Moore Smith, M.B., Ch.B. (Aberdeen), of Plumstead, to carry out part of the duties at a salary of £100 per annum, whilst Dr. Boulter is still to continue to visit the institution, retaining the salary hitherto paid by the Board for his services.

